# Tumor tagging: making cold tumors visible to immunotherapy

**DOI:** 10.3389/fimmu.2026.1981961

**Published:** 2026-10-28

**Authors:** Tong-Yun Wang, Yue Sun, Feng Cong, Yan-Dong Tang

**Affiliations:** 1 College of Animal Science and Technology, Zhongkai University of Agriculture and Engineering, Guangdong Engineering Technology Research Center of Biosafety and Intelligent Control for Aquatic Animals Diseases, Guangzhou, China; 2 State Key Laboratory of Animal Disease Control and Prevention, Harbin Veterinary Research Institute, Chinese Academy of Agricultural Sciences, Harbin, China; 3 Guangdong Hengqin Newcastle disease virus (NDV) Biotechnology Co., Ltd., Zhuhai, China

**Keywords:** cancer immunotherapy, cold tumors, immune recognition, mRNA vaccines, oncolytic viruses, tumor tagging

## Abstract

Cold tumors are poorly recognized by host immunity. Tumor tagging strategies—neoantigen, xenogeneic, and viral antigen tagging—endow tumor cells with identifiable antigens via engineered probiotics, oncolytic viruses, and mRNA delivery. These tactics yield compelling preclinical and clinical results, emerging as a novel promising strategy for cancer immunotherapy.

## Cold tumors are poorly recognized by anti-tumor immunotherapy

Anti-tumor immunotherapy has emerged as a transformative pillar of modern cancer care, encompassing immune checkpoint inhibitors (ICIs, see Glossary), chimeric antigen receptor T-cell (CAR-T) therapy, oncolytic virotherapy, and other innovative platforms. Despite remarkable advances, immunotherapies remain severely limited by intrinsic resistance, especially in patients harboring immunologically “cold” tumors. Cold tumors are typified by scarce intratumoral CD8^+^ T-cell infiltration, low tumor mutational burden, impaired antigen presentation, and a profoundly immunosuppressive tumor microenvironment (TME) (1). Together, these traits render tumor cells nearly “invisible” to host immunity and drive broad therapeutic resistance. Thus, novel strategies that overcome immune ignorance and reawake anti-tumor immunity are highly desirable. Emerging evidence has highlighted tumor tagging as a powerful approach that enhances tumor immunological visibility and profoundly remodels the TME, converts “cold” tumors into immunologically “hot” lesions, and elicits robust and durable anti-tumor immune responses. Here, we provide a concise, forward-looking overview of state-of-the-art tumor tagging strategies, discuss their translational implications, and highlight how these approaches are reshaping the therapeutic paradigm for immunologically cold solid tumors.

## The concept and biological rationale of tumor tagging

Tumor tagging can be defined as the intentional placement of antigenic signals into tumors to increase their recognizability by the immune system. To date, tumor tagging strategies directed against tumors have yielded compelling preclinical and clinical findings. Here, we highlight three major classes of strategies advanced in recent years: neoantigen tagging, xenogeneic antigen tagging, and viral antigen tagging. Of note, tumor tagging is not merely a discrete technology, but rather a unifying design principle that can be deployed through diverse delivery platforms, including engineered probiotics, engineered oncolytic viruses (OVs), and mRNA-based systems. In the sections below, we discuss these approaches in detail.

### Neoantigen tagging

Neoantigens serve as pivotal tumor-specific targets for precise cancer recognition and eradication, with therapeutic strategies encompassing personalized neoantigens tailored to individual mutational landscapes and public/shared neoantigens targeting recurrent driver mutations (e.g., *KRAS*, *VHL*, *PBRM1*) that enable off-the-shelf vaccine development (**Figure 1A**) (2–4). Neoantigens are efficiently delivered via multiple platforms including synthetic long peptides, mRNA (5), DNA, viral vectors (6, 7), and engineered probiotic vectors; among these, engineered probiotics enable tumor-targeted colonization, *in situ* neoantigen synthesis and release, concurrent activation of antigen-presenting cells, and remodeling of the immunosuppressive TME, thereby markedly enhancing immunogenicity (8). Preclinically, probiotic-based neoantigen vaccines elicit robust neoantigen-specific CD4^+^ and CD8^+^ T cell responses, drove profound TME reprogramming (reduced Tregs, myeloid-derived suppressor cells, and inhibitory macrophages), and achieved potent control or complete regression of primary and metastatic solid tumors in CT26 colorectal cancer and B16F10 melanoma models (8). In clinical trials, a phase I study of personalized synthetic long-peptide neoantigen vaccines in patients with resected high-risk clear cell renal cell carcinoma (ccRCC; NCT02950766) demonstrated 100% immunogenicity with favorable safety (2). All patients mounted robust vaccine-specific T cell responses, dominated by polyfunctional CD4^+^ memory T cells, and 7 out of 9 patients developed T cell reactivity against autologous tumor cells (2). Notably, the vaccine effectively targeted canonical ccRCC driver mutations including *VHL*, *PBRM1*, *BAP1*, *KDM5C*, and *PIK3CA*, with driver neoantigens showing significantly higher immunogenicity than passenger mutations (2). Collectively, these findings validate the robust translational potential of neoantigen-based strategies in precision cancer immunotherapy. Although personalized neoantigen tagging achieves impressive immunogenicity in early-phase clinical trials, several practical barriers persist. First, “turn-around time and cost” for whole-exome sequencing, neoantigen prediction, peptide/mRNA synthesis restrict broad deployment for personalized formats. Second, accurate neoantigen prioritization remains difficult; many predicted neoantigens fail to elicit tangible T-cell responses. Shared public neoantigen platforms partially bypass patient-tailored workflows but are limited by the low frequency of target driver mutations across patient cohorts. For probiotic-delivered neoantigen systems, inter-patient gut microbiome variability alters tumor colonization efficiency of engineered bacteria, and pre-existing anti-bacterial immunity may dampen therapeutic performance. In addition, safety risks including off-target microbial expansion require rigorous monitoring for *in-vivo* application.

**Figure 1:**
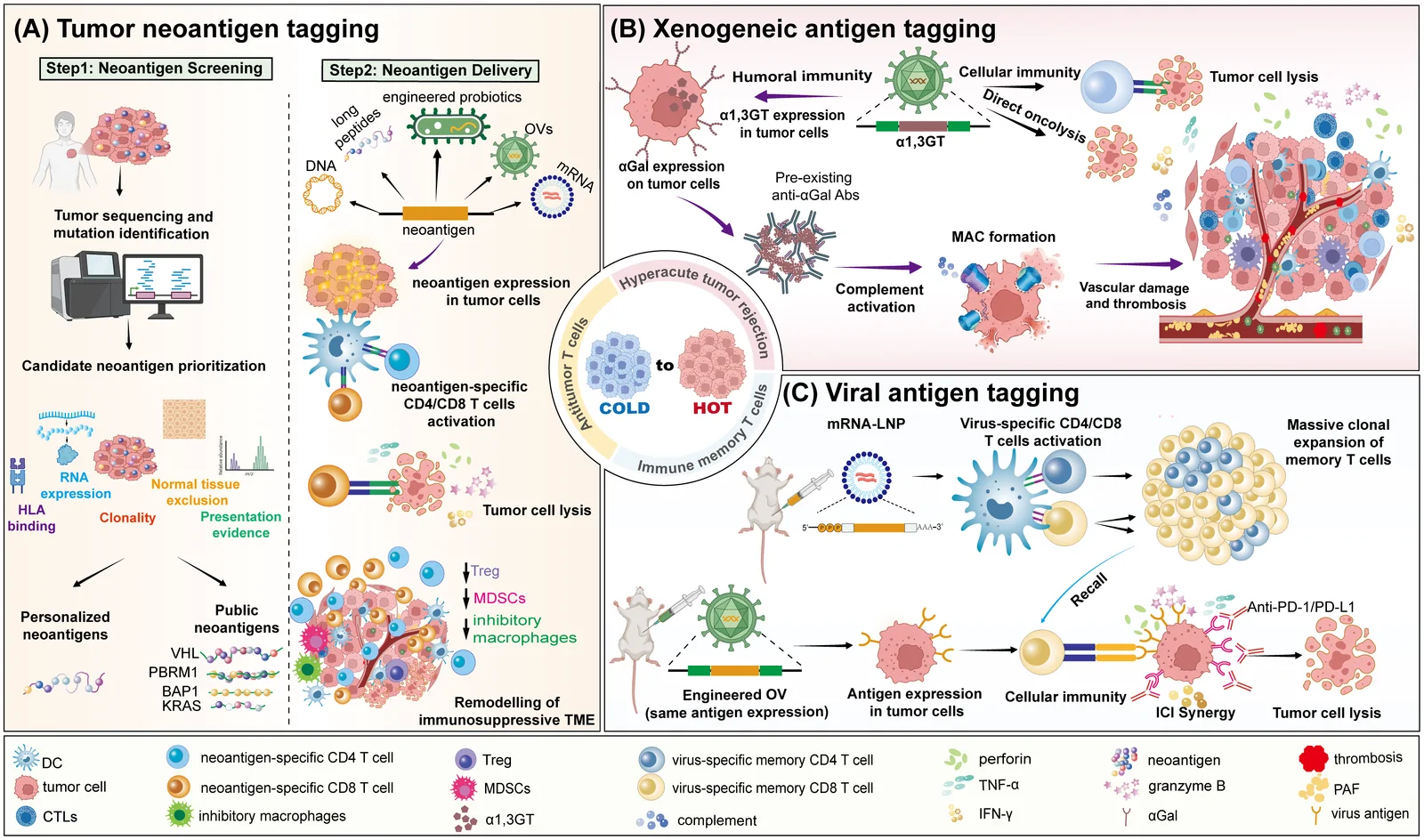
Tumor-tagging strategies making cold tumors visible to immunotherapy. Three core tumor-tagging paradigms for immunotherapy: **(A)** Neoantigen tagging. Tumor sequencing and mutation profiling enable the identification and prioritization of candidate neoantigens. Selected neoantigens delivered by long peptides, DNA, mRNA, engineered probiotics, or oncolytic viruses, thereby enabling direct antigen delivery and/or intratumoral neoantigen expression, promoting antigen presentation, activation of neoantigen-specific CD4^+^ and CD8^+^ T cells, tumor cell lysis, and remodeling of the immunosuppressive tumor microenvironment. **(B)** Xenogeneic antigen tagging. Oncolytic virus-mediated delivery of α1,3GT induces *de novo* αGal expression on tumor cells, triggering recognition by pre-existing anti-αGal antibodies and downstream effector responses, including complement activation, membrane attack complex formation, vascular injury, and thrombosis, culminating in hyperacute rejection-like tumor destruction. **(C)** Viral antigen tagging. mRNA-LNPs or engineered oncolytic viruses deliver viral antigens into tumors and recall or amplify pre-existing virus-specific memory CD4^+^ and CD8^+^ T cell responses, resulting in clonal expansion, enhanced cellular immunity, and tumor cell killing, while also creating opportunities for synergy with immune checkpoint blockade. Together, these approaches reshape the tumor microenvironment and support more effective immune-mediated tumor control.

### Xenogeneic antigen tagging

Tagging tumors with xenogeneic antigens represents a transformative immunotherapeutic strategy that repurposes evolutionary immunity for precision cancer treatment (9). This approach exploits the *α*1,3-galactosyltransferase (*α*1,3GT)–αGal–anti-Gal axis—a pathway uniquely absent in humans but highly active in most mammals—to redirect preformed natural immunity against tumors. Humans carry anti-αGal IgM/IgG antibodies generated by gut microbial stimulation, while lacking functional *α*1,3GT and thus unable to synthesize αGal epitopes. This evolutionary gap enables engineered oncolytic Newcastle disease virus (NDV-GT) to selectively deliver porcine α1,3GT into tumor cells, forcing *de novoα*Gal expression exclusively on malignant cells without modifying normal tissues. In infected tumor tissue, αGal decoration triggers a localized hyperacute rejection-like cascade: anti-αGal antibodies rapidly bind tumor vasculature and cell surfaces, activating complement-dependent cytotoxicity (CDC) and antibody-dependent cellular cytotoxicity (ADCC), inducing platelet-activating factor (PAF) release, fibrinogen deposition, and selective intratumoral microthrombosis (**Figure 1B**) (9). This dual vascular occlusion and immune cytotoxicity rapidly starve tumors of blood supply while amplifying innate and adaptive anti-tumor immunity. Preclinically, NDV-GT achieved complete tumor regression in CRISPR-engineered primary hepatocellular carcinoma cynomolgus monkeys, with safety confirmed in non-human primates showing no organ damage, minimal neutralizing antibody induction, and preserved viral efficacy (9). In a phase 1 interventional trial (ChiCTR2000031980) of 20 heavily pretreated patients with refractory metastatic solid tumors (including liver, ovarian, cervical, lung, esophageal, rectal, breast cancer and melanoma), intravenous NDV-GT achieved a 90.00% disease control rate (DCR), with 1 case of CR, 6 cases of PR, 11 cases of SD, and 2 cases of PD. Durable responses exceeding 6–36 months were observed in several subjects (9). Treatment was well-tolerated with only mild adverse events and no grade ≥3 toxicities, cytokine release syndrome, or neurotoxicity. Mechanistically, NDV-GT suppressed the Akt-IKK-NF-κB pathway to promote tumor apoptosis, while enhancing CD4^+^/CD8^+^ T-cell infiltration, dendritic cell maturation, and IFN-γ/TNF-α secretion to reverse immunosuppression (9). This xenogeneic antigen-tagging platform provides a clinically validated, safe, and broadly applicable strategy to convert “cold” tumors “hot” and enable systemic oncolytic virotherapy for diverse advanced malignancies. This approach leverages pre-existing circulating anti-αGal antibodies and does not depend on neoantigen identification. Still, translational obstacles remain. First, inter-individual variation in endogenous anti-αGal antibody titers can shape treatment efficacy; patients with low anti-αGal levels may exhibit attenuated complement-dependent and ADCC-mediated tumor damage. Second, intrapatient tumor heterogeneity creates variable viral infection efficiency across distinct tumor lesions; poorly infected tumor foci escape αGal antigen decoration and thereby evade immune attack. Third, although current phase-1 data show mild safety profiles, widespread intratumoral microthrombosis raises theoretical risks for systemic coagulation complications when treating large tumor burdens, which requires careful dose-escalation and patient stratification in further clinical studies.

### Viral antigens tagging

Viral antigen tagging capitalizes on population-wide pre-existing immunity elicited by routine vaccinations or prior viral infections, which establishes abundant pools of stable, high-affinity memory CD8^+^ T cells capable of naturally infiltrating tumors (10–12). This strategy stands out as a uniquely scalable, cost-effective, and broadly applicable antigen-tagging approach that circumvents the requirement for personalized neoantigen identification, synthesis, and validation. Clinically, this approach can be implemented through two translatable modalities: intratumoral or intravenous administration of engineered OVs, and therapeutic mRNA vaccines encoding target viral antigens (**Figure 1C**). Preclinical investigations have robustly validated this concept across diverse vector platforms: the H-TVAC (Hepatitis B tumor vaccine antigen complex) system utilizes HBsAg (Hepatitis B virus surface antigen)-expressing oncolytic vaccinia virus to redirect hepatitis B vaccine-elicited memory T cells against solid tumors (12); vesicular stomatitis virus (VSV) and single-cycle adenovirus vectors encoding SARS-CoV-2 Mem or Spike antigens direct high-affinity T cell immunity into established melanoma and colorectal tumors (13). Beyond activating memory CD8+ T cells, viral antigen tagging also mobilizes bystander T (T_BYS_) cells within the TME. Emerging evidence indicates that tumor-irrelevant T_BYS_ cells are abundant in the TME. To harness such T_BYS_ cells for tumor eradication, oncolytic viruses encoding T_BYS_ epitopes have been engineered to redirect pre-existing T_BYS_ cells toward tumor cells, thereby driving potent tumor suppression in multiple preclinical models (14). Accordingly, engineered OVs encoding lymphocytic choriomeningitis virus (LCMV) or SARS-CoV-2 epitopes selectively engage T_BYS_ cells to drive potent tumor elimination (14, 15). These alterations foster robust synergy with chemotherapy and PD-1/PD-L1 immune checkpoint blockade, significantly prolonging survival in preclinical models of melanoma, colorectal cancer, and triple-negative breast cancer (15).

Notably, this strategy demands no patient-specific customization, as most individuals harbor long-lived memory immunity to common viruses—including influenza, SARS-CoV-2, LCMV, and hepatitis B virus—arising from natural infection or universal vaccination (**Figure 1C**) (10–12). Together, harnessing pre-existing antiviral immune memory through OV- or mRNA-mediated viral antigen tagging creates a universal, off-the-shelf platform that retrains the immune system to target cancers, resolving long-standing obstacles in the clinical translation of personalized immunotherapy and oncolytic virotherapy. This off-the-shelf concept relies on pre-established antiviral memory T-cell pools within individual patients. The major limitation lies in “inter-patient heterogeneity of memory repertoires”: history of infection or vaccination determines the magnitude and quality of available virus-specific memory T-cells. Patients lacking corresponding antiviral memory cannot benefit from a given viral-antigen tagging modality. Moreover, pre-existing neutralizing antibodies against oncolytic viruses can reduce vector delivery to tumor sites. For bystander TBYS-cell-oriented tagging approaches, the abundance and functional status of tumor-resident irrelevant memory T-cells vary greatly across tumor types and patients, limiting predictable therapeutic output.

## Tumor-intrinsic defects in antigen processing and presentation represent a major stratification biomarker for tumor-tagging therapies

All three tumor-tagging modalities depend partially or fully on antigen processing and presentation machinery, yet tumors frequently harbor genetic or epigenetic defects in the MHC-I antigen-presentation pathway (e.g., loss-of-function mutations in β2M, downregulation of HLA class-I molecules, impairment of the proteasome-processing cascade) (16, 17).

For neoantigen tagging: When neoantigens are presented via MHC-I on tumor cells themselves, β2M/HLA-I loss abrogates direct recognition by CD8^+^ T cells. Nevertheless, delivery platforms such as probiotics, oncolytic viruses and mRNA can drive abundant neoantigen uptake by host professional antigen-presenting cells (APCs, e.g., dendritic cells). Cross-presentation by intact host APCs can retain anti-tumor reactivity even when tumor cells are MHC-I-deficient, yet the magnitude of cross-presentation varies and rarely fully rescues complete APP loss in tumor parenchyma.

For xenogeneic αGal antigen tagging: This strategy is comparatively less vulnerable to tumor-cell-intrinsic APP defects. Its primary antitumor mechanisms are humoral-driven: anti-αGal antibody binding, complement activation, ADCC and local microvascular thrombosis do not require tumor-cell MHC-I-dependent antigen presentation. Adaptive T-cell responses induced secondary to *in-situ* tumor cell death can still be blunted if tumor lesions lack MHC-I, but the core therapeutic effect can persist independent of tumor-cell APP competence. This represents a unique advantage for treating APP-deficient cold tumors.

For viral antigen tagging: Redirecting pre-existing virus-specific CD8^+^ T cells demands MHC-I expression on tumor targets to recognize displayed viral epitopes. Severe tumor-intrinsic MHC-I loss will therefore abolish direct killing by these memory T-cells. Of note, oncolytic virus infection can trigger immunogenic cell death and recruit functional host APCs; cross-presentation of tumor-associated neoepitopes may still occur, yet this is a secondary, collateral effect rather than the primary mechanism of viral-antigen tagging. Thus, profound MHC-I/APP defects largely negate the core activity of viral antigen tagging.

Collectively, tumor-intrinsic antigen-presentation defects represent a major stratification biomarker for tumor-tagging therapies. Xenogeneic antigen tagging is more resilient to such defects, while neoantigen- and viral-antigen tagging are substantially compromised upon complete loss of tumor-cell MHC-I function. Patient stratification based on tumor MHC-I status will be critical for future clinical implementation.

## Concluding remarks

Tumor tagging is notable because it introduces a set of controllable variables in immune visibility. However, there are several bottlenecks in this field. First, how much tagging is enough? A threshold issue almost certainly exists: too little expression may fail to engage immunity, whereas excessive or poorly localized expression may increase off-tumor risk or intensify anti-vector responses. Second, delivery platforms may exhibit tumor-type-dependent activity; thus, distinct malignancies require tailored optimization of delivery systems. Third, host immunity is both an asset and a constraint. Pre-existing antibodies, memory repertoires, and anti-vector responses may all shape efficacy, toxicity, and repeat-dosing feasibility. Fourth, rational combination design is indispensable. The further development of tumor tagging relies on clarifying the rational application scenarios for individualized and off-the-shelf strategies, including monotherapy, sequential administration and combinatorial regimens.

Overall, tumor tagging offers a viable framework to render cold tumors responsive to immunotherapy. By delivering neoantigens, xenogeneic or pathogen-derived antigens to tumor tissues, this approach overcomes insufficient immune recognition—a key bottleneck of cancer immunotherapy. Though still in early development, emerging evidence indicates tumor tagging can complement mainstream immunotherapies. Future research should focus on defining clinically applicable conditions, with optimized antigen delivery, expression dynamics, spatial distribution, combination regimens and patient selection. Addressing these challenges will enable tumor tagging to expand immunotherapy efficacy in solid tumors by targeting and enhancing tumor immune recognition.

## Data Availability

The original contributions presented in the study are included in the article/supplementary material. Further inquiries can be directed to the corresponding authors.

## Glossary

ADCC: antibody-dependent cellular cytotoxicity, an immune effector mechanism whereby Fc receptor–bearing innate immune cells recognize antibody-coated tumor cells and trigger target cell apoptosis
α1,3GT: α1,3-galactosyltransferase, a glycosyltransferase that synthesizes αGal epitopes; functionally inactive in humans but conserved in most mammals
αGal: α1,3-galactosyl epitope, a xenogeneic glycan absent in humans. Ectopic expression on tumor cells elicits hyperacute rejection–like anti-tumor responses via preexisting anti-αGal antibodies
CAR-T cell: chimeric antigen receptor T-cell, genetically engineered T lymphocytes expressing a synthetic tumor-targeting receptor that mediates MHC-independent tumor cell recognition and lysis
CDC: complement-dependent cytotoxicity, a humoral immune pathway in which antibody binding activates the complement cascade, forming membrane attack complexes that directly lyse tumor cells
engineered probiotics: genetically modified commensal bacteria designed to selectively colonize tumors, express therapeutic antigens, remodel the tumor microenvironment, and enhance anti-tumor immune responses
ICIs: immune checkpoint inhibitors, a monoclonal antibodies that block inhibitory immune checkpoints (e.g., PD-1, PD-L1, CTLA-4) to reverse T-cell exhaustion and restore anti-tumor immunity
oncolytic virotherapy: a therapeutic approach that uses engineered or natural viruses to selectively replicate in and lyse tumor cells, while triggering systemic anti-tumor immunity and remodeling the immunosuppressive TME
OVs: oncolytic viruses, viruses engineered to selectively replicate in and lyse tumor cells, trigger immunogenic cell death, and remodel the tumor microenvironment
PAF: platelet-activating factor, a potent phospholipid mediator that induces platelet aggregation, vascular permeability, and local thrombosis, contributing to tumor vascular disruption during xenogeneic antigen tagging
PD-1/PD-L1: programmed death-1/programmed death ligand-1, key immune checkpoint molecules that suppress T-cell activation and induce exhaustion; major targets for cancer immunotherapy
Personalized neoantigens: patient-specific tumor antigens derived from unique somatic mutations in individual patientsused for customized cancer vaccine development
Public/shared neoantigens: recurrent tumor antigens derived from conserved driver mutations (e.g., KRAS, VHL, PBRM1) across patients, enabling off-the-shelf cancer vaccines
TBYS cells: tumor-irrelevant bystander T cells, tumor-infiltrating T cells with non-tumor specificity that exert bystander anti-tumor cytotoxicity in response to inflammatory signals within the TME
tumor tagging: a unifying immunotherapeutic strategy that introduces identifiable antigenic signals into tumor tissues to enhance immune recognition, convert cold tumors hot, and restore responsiveness to immunotherapy
TME: tumor microenvironment, the dynamic cellular, vascular, metabolic, and immunological milieu surrounding tumor cells, typically characterized by immune suppression and therapy resistance
